# The Multicomponent Anthropometric Model for Assessing Body Composition in a Male Pediatric Population: A Simultaneous Prediction of Fat Mass, Bone Mineral Content, and Lean Soft Tissue

**DOI:** 10.1155/2013/428135

**Published:** 2013-03-11

**Authors:** Dalmo Machado, Sérgio Oikawa, Valdir Barbanti

**Affiliations:** ^1^School of Physical Education and Sport of Ribeirao Preto, University of Sao Paulo, Ribeirão Preto, Brazil; ^2^Department of Statistic, Faculty of Science and Technology, Paulista State University, Presidente Prudente, Brazil

## Abstract

The aim of this study was to propose and cross-validate an anthropometric model for the simultaneous estimation of fat mass (FM), bone mineral content (BMC), and lean soft tissue (LST) using DXA as the reference method. A total of 408 boys (8–18 years) were included in this sample. Whole-body FM, BMC, and LST were measured by DXA and considered as dependent variables. Independent variables included thirty-two anthropometrics measurements and maturity offset determined by the Mirwald equation. From a multivariate regression model (*Ymn* = *x*(*r* + 1)(*r* + 1)*nβ*_*m*_ + *εnm*), a matrix analysis was performed resulting in a multicomponent anthropometric model. The cross-validation was executed through the sum of squares of residuals (PRESS) method. Five anthropometric variables predicted simultaneously FM, BMC, and LST. Cross-validation parameters indicated that the new model is accurate with high *R*_PRESS_^2^ values ranging from 0.94 to 0.98 and standard error of estimate ranging from 0.01 to 0.09. The newly proposed model represents an alternative to accurately assess the body composition in male pediatric ages.

## 1. Introduction

Estimate body composition of children is not an easy task, since the relationships between body components during growth are not constant as in adults. Anthropometric-based equations remain an adequate alternative for determining the body composition of pediatric populations in field settings. However, the advent of new technologies has enabled new ways for body composition assessment, thus, rendering the traditional anthropometry inaccuracy as a representative standard [1]. There are some methodological concerns when using the current anthropometric models: several equations have been developed using a two-compartment model (2C model) either using hydrostatic weighing [2, 3] or other densitometric techniques; however, this approach relies on assumptions, specifically concerning the fat-free mass (FFM) density (1.1 g/cc) and hydration (73.2% of total-body water within the FFM) that, although stable for adults, may vary substantially during growth. In fact, from childhood through adolescence, total-body water (TBW) decreases whereas bone mass increases which means that FFM density is lower than 1.1 g/cc, at younger ages, approaching that value when chemical maturity is reached [4]. Therefore, 2C models tend to overestimate FM and underestimate FFM in children, and their use as a criterion method for developing anthropometric-based models is inaccurate. For that reason, the use of 3C and 4C compartment models are preferred for determining the body composition in children [4], since fewer assumptions are used as more FFM components are measured.

The advent of dual-energy X-ray absorptiometry (DXA), measures of FM, bone mineral content (BMC), and lean soft tissue (LST) are obtained. Hence, DXA can be considered as a 3C model since the estimates of three components are obtained as follows: first by separating pixels into those with soft tissue only (FM plus LST) and those with soft tissue plus BMC, based on two different photon energies (lower and higher energies, resp.) [5]. The DXA provides precise [6, 7] and accurate [8–11] measures of FM and FFM (as LST plus BMC) when compared to multicompartment models. In addition, given its low risk and quick assessment, the DXA use has been implemented in large multicenter studies, including the National Health and Nutrition Examination Survey [12].

However, the availability of DXA in the clinical and fields settings is limited given its cost. Therefore, simple solutions are required for estimating body composition in children and anthropometric parameters, such as skinfolds and circumferences, which have been widely used as bedside techniques in different contexts. Thus, the aim of this study was to develop and cross-validate multicomponent-anthropometric-based equations to simultaneously estimate FM, BMC, and LST in a male pediatric population, using DXA as the criterion method. 

## 2. Methods

### 2.1. Study Population

The study followed a cross-sectional design, consisting of a sample of 408 young males between 8 and 18 years of age. The subjects were recruited voluntarily from a population of students that could be engaged in systematic programs of sports, or not, considered as athletes and nonathletes, respectively. The athletes came from sports centers (*n* = 177) and nonathletes from schools (*n* = 231). Children with a regular sports practice were engaged in soccer field (*n* = 143), athletics (*n* = 11), football court (*n* = 20), and judo (*n* = 3). The nonathletes came from public (*n* = 142) and private (*n* = 89) school. Medical examinations were conducted to assure that children were healthy and not taking medications that could affect metabolism, appetite, or growth. The number of White participants was relatively higher (*n* = 270) compared to Blacks (*n* = 79), Hispanics (*n* = 50), and Asians (*n* = 9), classified by race self-declared. This sample comes from a large ethnic mixture and previous analysis that showed no statistical differences in interracial body composition (data not shown), so, the final samples (*n* = 408) were considered as uniform. To determine the sample size, we followed the Bolfarine and Bussab [13] recommendation, and based on a pilot analysis with subjects presenting a large variance in the dependent variable (FM), the estimation of the desired error (1.25%) and confidence interval (95%) determined that at least 300 subjects would be required.

The study followed the guidelines and regulations of directing human research, and agreements were obtained from the parents or guardians to all procedures. The approval was granted by the Ethics in Research Department of the School of Physical Education and Sport, University of São Paulo (CEP332007/EEFE/04.04.2007-2006/32), which also adhere to the Helsinki Declaration.

### 2.2. Study Protocol

Each subject was evaluated in the laboratory, in the morning after an overnight fast, in a single session, and always by the same examiner, and all measurements, were performed during a period of three months. Before the measurements the subjects were asked to empty their bladders. Dressed in shorts and shirt, the total-body DXA examination was applied using the system for total-body scan, according to manufacturer's guidelines. The anthropometric measures were performed according to the literature recommendations [14, 15], summarized below. 

#### 2.2.1. The Dependent Variables: Dual-Energy X-Ray Absorptiometry

Whole and regional body composition was estimated with a DXA Scanner Lunar DPX-NT (GE Medical, Software Lunar DPX enCORE 2007 version 11.40.004, Madison, WI). The software identified the physical characteristics of ethnicity, gender, and age and automatically adjusted the scan mode, speed, and images resolution. 

Body weight was determined from DXA, and the dependent variables of interest were fat mass (FM, kg), bone mineral content (BMC, kg), and lean soft tissue (LST, kg).

#### 2.2.2. The Independent Variables

(1) *Anthropometrics. *The subject body mass, height, and seating height [15] were measured with a digital scale (Filizola, PL 200, Campo Grande, MS) and a fixed wall stadiometer (Sanny Professional-ES2020, São Paulo, SP), respectively. The skinfolds (biceps, triceps, subscapular, chest, midaxillary, suprailiac, vertical abdominal, horizontal abdominal, mid-thigh, and medial calf), circumferences (chest, relaxed arm, contracted arm, forearm, wrist, waist, abdominal, hip, proximal thigh, and calf), and breadths (biacromial, biiliac, chest, elbow, bitrochanteric, wrist, knee, and bimalleolar) were measured by conventional procedures stated in the literature [15, 16] using Sanny scientific equipment.

(2) *Maturation. *For determining the biological development, the maturity offset was predicted by gender-specific regression equations based upon noninvasive techniques, using chronological age, height, body mass, sitting height, and leg length measurements [17]. The method predicts years from peak height velocity (PHV) according to the Mirwald et al. [17] equation for boys:

(1) PHV=−9.236+0.0002708(Lh×Sh)−0.001663(A×Lh)+0.007216(A×Sh)+0.02292(Wt/Ht×100),

where Lh stands for legs height (cm), Sh for seating height (cm), A for age (years), Wt for body weight (kg), and Ht for height (cm).

(3) *Chronological Age. *Chronological ages were based on birth year and grouped in decimal values adjusted to the nearest integer.

To ensure the precision of the results, intra evaluator technical errors of measurement absolute (TEM) and relative (TEM%) were calculated (Table 1). In subsequent days, duplicates for all measures were applied in thirteen subjects, when the results were always within the expected tolerance limits [15].

### 2.3. Statistical Analysis

The SPSS Statistics, version 13, for Windows (SPSS Inc., Chicago, IL) was used to analyze the data of descriptive statistics (mean, standard deviation, range, technical error of measure relative, absolute, and the confidence interval—CI 95%) were used to describe the sample, and correlation coefficient was applied to verify the basic assumption of the relations between dependent and independent variables. For developing the multicomponent anthropometric equation, a multivariate regression model (_*n*_ *Y*_*m*_ = _*n*_ *X*_(*r*+1)(*r*+1)_ *β*_*m*_ + _*n*_ *ε*_*m*_) was utilized as diagonal mutual analysis, parameter estimation, and the least squares errors method by *R*-Free Software [18]. When the choice of remaining variables the following criteria were used (a) maintenance of a high correlation between independent and dependent variables, (b) uniformity of the data, (c) centralized distribution of the residuals, (e) reducing the number of independent variables while maintaining the highest levels of significance after stepwise, with adjustments by the Pillai approach to test the *F* values, (f) multicollinearity tolerated, (g) determining the *β* values in a multivariate model, and (h) remaining of the high precision and validity of the final model. More explanations of the multivariate analysis are given by Johnson and Wichern [19].

For performing the validation of the models we used thePRESS statistic [20]. From the deletion of an observation, proposed equations with the remaining sample are conducted, and the process is repeated. The PRESS statistic is defined as the sum of squares of residuals (PRESS) in:

(2) $$\begin{array}{c} \text{ PRESS }=\sum_{i=1}^{n}{\left[y_{i}-{\hat{y}}_{\left(i\right)}\right]}^{2}. \end{array}$$

Thus, a model with a high degree of predictability for excluded observations gives the value of the *R*_PRESS_^2^ (close to 1) and a standard estimated error (SEE_PRESS_) near zero. In summary, the PRESS statistic gives an indication of the predictive ability of the regression model. The validation procedure that uses PRESS is similar to the application of the equation to an independent sample [21].

## 3. Results

Characteristics of the total sample are shown in Table 1, including range (minimum–maximum), TEM, TEM%, and confidence interval (95%).

Table 2 presents the correlation matrix within some of the 32 independents, including size measures, skinfolds, circumferences, breadths, and maturation by PHV with and the dependent variables.

**Figure 1:**
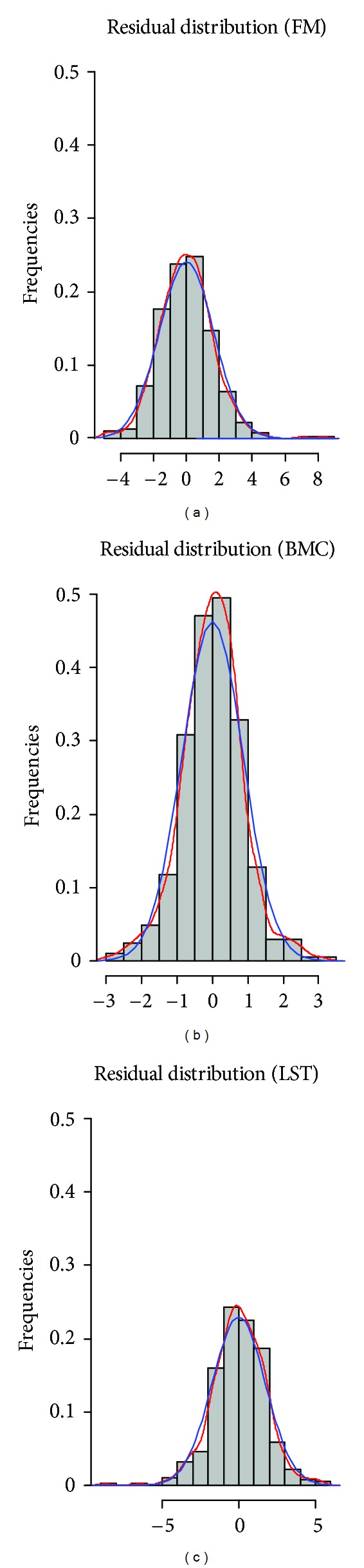
Multivariate distribution of residuals for fat mass (FM), bone mineral content (BMC), and lean soft tissue (LST).

A centered distribution of the residuals (differences) was observed for the response components (Figure 1).

From all 32 initial variables used as predictors of the dependent variables, a stepwise regression was performed individually for FM, BMC, and LST in order to select the common variables for all three components, with the higher significance level. The number of predictor variables was reduced after 27 eliminations, and a final model was obtained with five independent variables and high precision (*R* ^2^), meaning that the models largely explained the variance of the dependent variables (Table 3). Here, the Pillai method approach was used to test the *F* values. The estimated parameters vector (*β*) of the model was obtained for each variable, resulting in a single model for all dependent variables (Table 3).

From the multivariate parameters, it was possible to predict simultaneously each body component (FM, BMC, and LST), considering the interrelationship of dependent variables, unlike the traditional methods (one-dimensional analysis). Multicollinearity within the final independent variables was tested, and cases were found in which the variables were highly collinear. In those cases, an independent variable in the model was eliminated and performed the ratio between the largest and the lowest eigenvalues [20], until resulting in a final product with only moderate multicollinearity (*λ* = 167.0637).

Table 4 summarizes mean values and standard deviations for the descriptive characteristics obtained from the DXA scan by age group. The FM showed increases up to the age of 13, which tend to stabilize. However, there were no statistically significant age differences. All other significant differences for subsequent ages in BMC were found from 11- to 12-year-old age group (*P* = 0.021), from 13- to 14-year-old age group (*P* = 0.001), and from 14- to 15-year-old age group (*P* = 0.001); for LST, the differences were found from 11- to 12-year-old age group (*P* = 0.001), from 13- to 14-year-old age group (*P* = 0.001), and from 14- to 15-year-old age group (*P* = 0.001).

### 3.1. The Precision of the Model

**Figure 2:**
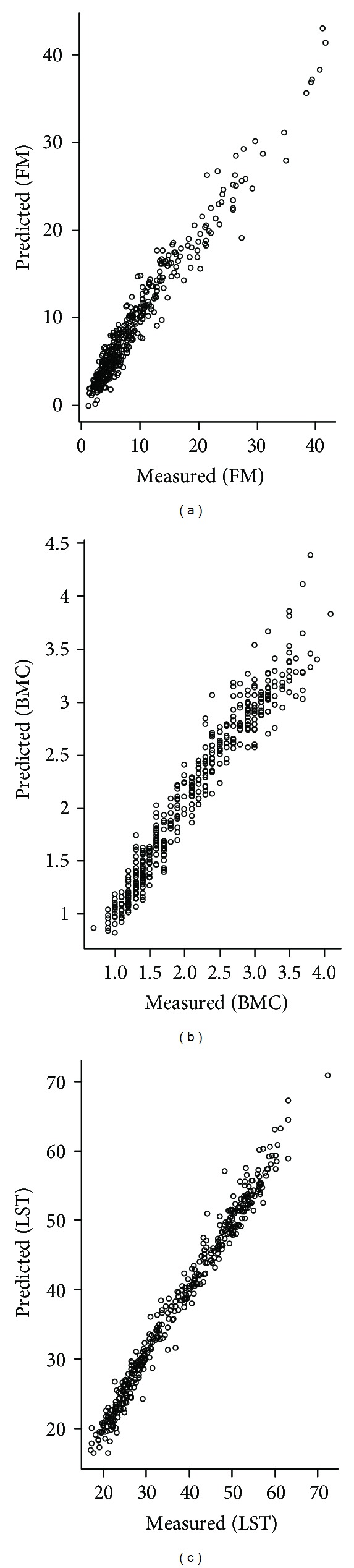
Scatterplot of predicted and actual fat mass (FM), bone mineral content (BMC), and lean soft tissue (LST) values in the male pediatric population.

The correlations between the predicted values (of the model) and those observed (by DXA) in FM, BMC, and LST (Figure 2) showed an increased dispersion at higher scores of body composition.

The PRESS related statistics (*R* ^2^), adjusted coefficients of determination (Adj  *R* ^2^), and standard error of estimate (SEE_RESIDUAL_) for residual analysis are showed in Table 3.

### 3.2. Cross-Validation

In this study, the error was determined by the outcome of *Y*-observed minus *Y*-estimated. Parameters of internal validation included *R*_PRESS_^2^ statistic and SEE_PRESS_, as observed in Table 3. The model is valid according to the assumptions defined in the methodology, where *R*_PRESS_^2^ should be close to “1” and SEE_PRESS_ near “0”.

Then, the final model for each dependent variable could be expressed as

(3) FM=−  0.0857  Height+0.3139  weight   +0.1970  SkSi+0.2350  SkHab−0.6571  PHV,BMC=0.0032  Height+0.0392  weight   −0.0095  SkSi−0.0105  SkHab+0.0525  PHV,LST=0.0820  Height+0.6419  weight   −0.1964  SkSi−0.2321  SkHab+0.7047  PHV,

where SkSi stands for suprailiac skinfold (mm), SkHab for horizontal abdominal skinfold (mm), and PHV for peak height velocity (years).

## 4. Discussion

The multicomponent model approach presented in this study showed a high correlation in most comparisons between independent and dependent variables (Table 2), suggesting the possibility of using these variables as an alternative method.

The multicomponent determination of body composition during growth finds application in field and clinical settings allowing specific definition for the component of interest. In sports, for example, monitoring the training process to reduce FM or increase lean mass may be of interest to technicians, aiming to improve sports performance. For most cases, the uncertainty of which component has contributed to an increase in body weight may compromise an adequate decision for exercise prescription, since the true relationships between FM and FFM are not known. Therefore, an accurate and precise body composition estimation is required using simple methods [1].

In the present study, the greater associations of FM were observed with skinfolds, BMC, with growth components (height, weight, breadth, and PVC) and LST with growth components and circumferences (Table 2), expressing the real expected relationships between the types of measurements and the components measured in a combined prediction. This is a crucial fact to determine the robustness of the model [19]. This is so because the combined estimation of the parameters produces zero restrictions on coefficients of other equations [22]. The relationship between the predictors and the response variables must be strong. 

However, the robustness of the model can be compromised if there is multicollinearity between independent variables. The multicollinearity was examined, given the natural relationships between the independent variables. Therefore, the elimination of independent variables was required, and those who are not commonly used in the literature or without a high predictive significance were removed. Apart from being a practical model, the least number of possible variables should be considered. In this case, the estimates of regression coefficients become very sensitive to small changes in the planning matrix. The variations of the estimators are high, making testing of *H*_0_: *β*_*j*_ = 0 versus *H*_*A*_: *β*_*j*_ ≠ 0  diff = 0; therefore, important independent variables could mistakenly be removed. One of the assumptions of the linear model is that the rank of the matrix (*X*′*X*) is equal to *k* + 1. Thus, in addition to moderate multicollinearity (*λ* = 167.0637) and near the bottom of this classification (from 100 to 1000) [23] and the determinant away from zero, the rank axis of *X*′*X* matrix is complete. Then, there is its classical inverse (*X*′*X*)^−1^ [det (*X*′*X*) ≠ 0], multiplied by the right side of the normal equation system, allowing the *β* obtaining the least squares estimator. The classical inverse matrix procedure was calculated, resulting in the root close to the efficiency characteristic, once the issue of moderate multicollinearity was observed, near to lower limit. The gain in predictive efficiency in the use of multivariate analysis in relation to various regressions is well proven. Basically, this is true because the efficiency jointly estimates the parameters and produces zero restrictions on coefficients of other equations [20], with the same error as vectors of estimated betas, enhancing the prediction.

So far, only the FM has been predicted by pediatric anthropometric models, determined by anthropometric-based models which have been developed against densitometric techniques in children [2, 3, 24–26] showing relatively low ability in predicting the variability of the reference method (*R* ^2^ < 0.80) when compared to those developed from the present study (Table 3). However, investigations can be controversial when very young or very obese children are involved in the observations [27], and the literature expresses caution in the estimation of body composition when BMI is high [1, 28, 29]. The model proposed in this study was able to predict the body composition also of overweight subjects according to the Cole et al. [30] cutoff points (11 cases). Even if these cases were removed, the accuracy of the model remained similar, confirming a possible generalization of the predictive equations for assessing overweight children.

The method of internal validity adopted [20] confirmed the effectiveness of the model to predict the components of body composition with a high internal validity (*R*_PRESS_^2^ = 0.94 to 0.98) and low proportional errors of estimation (SEE_PRESS_ = 0.01 to 0.09), that is, a score *R*_PRESS_^2^ = 0.9490 for FM (Table 3) may explain about 94.90% of the variability in predicting new observations in independent samples, compared with about 98.08% of variability in the original data, explained by the least squares (*R* ^2^) method. Also, the high independent *R*_PRESS_^2^ (94.02% and 98.04%), respectively, for BMC and LST indicates the strength of the model in predicting the lean body composition of young males between 8 and 18 years of age. These results provide the generalizability of the model, even when the variance of body composition is high. The low dispersion of the measured and predicted values for the body components (Figure 2) seems to confirm this hypothesis.

To facilitate a better understanding of the practical utility of the model, we show the following example for predicting FM, BMC, and LST in a 13-year old boy (Table 5). After obtaining, the measures (independent variables) of height, weight, maturation (PHV), and skinfolds (suprailiac and horizontal abdominal) simply apply the anthropometric multicomponent matrix described in Table 3.

The products of each measure, multiplied by its *β* coefficient regression, result in absolute values (kg) for FM, BMC, and LST.

A limitation of this study is that although DXA was used as a reference method to develop our model, this technique is not considered the gold standard for pediatric populations. A four-compartment model (4C model) is actually the most strong model for accurately assesses body composition in children as it accounts for the variability of the main FFM components [31]. Though its use is recommended as criterion, this method is time-consuming and requires sophisticated equipment, specialized technicians, and high costs which make it difficult for use in large samples [32]. In addition, the 4C model is not free of errors, considering the number of required techniques necessary for determining the main FFM constituents (water and mineral) [8]. Therefore, the use of DXA is an alternative chosen by several investigators to develop predictive equations for children and adolescents [25, 33–38]. In fact, a recent study revealed DXA as a precise and valid method for body composition assessment [39]. Another limitation that needs to be addressed is the ethnical differences of this sample, who could limit the generalization of the equations to other populations. Therefore, further studies are recommended that examine the accuracy of the models before its application. 

Concluding, new anthropometric-based model for assessing body composition of children and adolescent males was proposed. Considering the unavailability of sophisticated instruments in field and clinical settings, these models proved to be a valid and alternative solution to estimate body composition in a male pediatric population.

## Figures and Tables

**Table 1:** Descriptive statistics of body composition in boys (*n* = 408), including absolute and relative TEM of the dependent (DXA) and independent measures (maturation, body size, and skinfolds).

	Range	Mean	SD	TEM	TEM%	CI 95%
DXA						
Fat mass (kg)	1.3–41.8	9.3	7.5	0.22	1.42	8.6–10.0
Bone mineral content (kg)	0.7–4.1	2.1	0.8	0.01	0.03	2.1–2.2
Lean mass tissue (kg)	17.1–72.6	38.1	12.7	0.06	0.15	36.9–39.4
Age/maturation/anthropometrics						
Age (year)	8–18	13.7	2.99	—	—	12.9–13.5
PHV (year)	−4.7–4.5	−0.5	2.5	—	—	−0.8–0.3
Seating height (cm)	61.5–99.5	82.3	8.8	0.26	0.30	81.4–83.1
Height (cm)	120.3–196.8	158.1	17.7	0.17	0.11	156.4–159.8
Weight (kg)	20.6–119.4	50.2	17.4	0.27	0.29	48.5–51.9
Suprailiac skinfold (mm)	2.8–64.5	13.3	10.2	0.35	2.27	12.4–14.3
Horizontal abdominal skinfold (mm)	1.5–66.0	16.5	12.0	1.59	4.96	15.4–17.7

TEM: absolute technical error of measurement; TEM%: relative technical error of measurement; CI: confidence interval; DXA: dual-energy X-ray absorptiometry; PHV: years for peak height velocity.

**Table 2:** Correlation matrix between independent and dependent variables in the pediatric population.

Independent													Dependent		
	Wt	SkTr	SkSi	SkHab	SkTh	CiAr	CiWs	CiTh	Br El	BrKn	PHV	Age	FM (kg)	BMC (kg)	LST (kg)
Ht	0.84	−0.13	0.12	0.11	−0.14	0.67	0.65	0.69	0.85	0.70	0.94	0.88	0.28	0.91	0.95
Wt		0.29	0.54	0.52	0.27	0.91	0.89	0.89	0.87	0.77	0.87	0.78	0.70	0.92	0.91
SkTr			0.85	0.86	0.89	0.44	0.46	0.37	0.11	0.23	−0.09	−0.17	0.82	−0.02	−0.10
SkSi				0.90	0.80	0.65	0.68	0.54	0.34	0.38	0.17	0.08	0.92	0.24	0.17
SkHab					0.83	0.62	0.66	0.56	0.31	0.35	0.15	0.05	0.92	0.22	0.14
SkTh						0.41	0.45	0.38	0.09	0.19	−0.09	−0.16	0.80	−0.02	−0.11
CiRa							0.89	0.86	0.77	0.67	0.74	0.66	0.75	0.77	0.76
CiWa								0.83	0.75	0.69	0.70	0.61	0.78	0.74	0.72
CiTh									0.76	0.71	0.74	0.65	0.69	0.79	0.77
BrEl										0.79	0.82	0.75	0.46	0.84	0.87
BrKn											0.67	0.60	0.50	0.73	0.72
PHV												0.97	0.32	0.93	0.95
Age													0.22	0.87	0.89

Ht: height; Wt: weight; Sk: skinfold; SkTr: triceps; SkSi: suprailiac; SkHab: horizontal abdominal; SkTh: mid-thigh; Ci: circumference; CiRa: relaxed arm; CiWa: waist; CiTh: proximal thigh; Br: breadth; BrEl: elbow; BrKn: knee; PHV: years for peak height velocity; FM: fat mass, BMC: bone mineral content; LST: lean soft-tissue.

**Table 3:** Multicomponent anthropometric model matrix, precision, and internal cross-validity for simultaneously measuring of body composition in boys.

	*β* FM	*β* BMC	*β* LST
Height (cm)	−0.0857	0.0032	0.0820
Weight (kg)	0.3139	0.0392	0.6419
SkSi (mm)	0.1970	−0.0095	−0.1964
SkHab (mm)	0.2350	−0.0105	−0.2321
PHV (yr)	−0.6571	0.0525	0.7047
Precision			
*R* ^2^	0.9808	0.9930	0.9981
Adj *R* ^2^	0.9805	0.9929	0.9981
SEE_residual_ (kg)	1.6660	0.1923	1.7480
Cross-validation			
PRESS	1162.433	15.37255	1280.083
*R*_PRESS_^2^	0.9490	0.9402	0.9804
SEE_PRESS_ (kg)	0.0850	0.0098	0.0892

*β*: estimated parameter vector; FM: fat mass, BMC: bone mineral content; LST: lean soft tissue; Sk: skinfold; SkSi: suprailiac; SkHab: horizontal abdominal; PHV: age for peak height velocity; *R* ^2^: coefficient of determination (observed and cross-predicted); Adj *R* ^2^: adjusted coefficient of determination; SEE_residual_: residual standard error of estimate; PRESS: sum of squares of residuals; *R* ^2^_PRESS_: press coefficient of determination; SEE_PRESS_: press standard error of estimate.

**Table 4:** Mean and standard deviation of DXA dependent variables by age group.

Age (years)	FM	BMC	LST
8 (*n* = 28)	6.1 ± 4.4	1.2 ± 0.2	22.0 ± 2.7
9 (*n* = 32)	6.6 ± 4.2	1.2 ± 0.2	23.3 ± 3.0
10 (*n* = 34)	7.0 ± 4.5	1.3 ± 0.2	24.5 ± 3.3
11 (*n* = 40)	7.8 ± 6.2	1.5 ± 0.3*	26.5 ± 3.8*
12 (*n* = 37)	8.4 ± 6.5	1.8 ± 0.3	32.3 ± 5.3
13 (*n* = 39)	10.9 ± 9.5	2.0 ± 0.4*	36.2 ± 6.3*
14 (*n* = 47)	10.3 ± 9.4	2.3 ± 0.5*	42.4 ± 7.1*
15 (*n* = 42)	11.1 ± 8.1	2.7 ± 0.5	48.4 ± 6.8
16 (*n* = 39)	10.3 ± 5.5	3.0 ± 0.4	50.6 ± 5.0
17 (*n* = 40)	11.9 ± 8.7	3.0 ± 0.4	52.0 ± 6.0
18 (*n* = 30)	10.1 ± 8.7	3.1 ± 0.8	53.8 ± 5.9

DXA: dual-energy X-ray absorptiometry; FM: fat mass; BMC: bone mineral content; LST: lean soft tissue. *Subsequent age significantly different at *P* < 0.05.

**Table 5:** A worked example for predicting fat mass (FM), bone mineral content (BMC), and lean soft tissue (LST) for a boy.

Variables	Measures	FM	Product	BMC	Product	LST	Product
Height (cm)	*148.3 *	***−*0.0857**	*−12.71 *	**0.0032**	*0.48 *	**0.0820**	*12.16 *
Weight (kg)	*40.0 *	**0.3139**	*12.56 *	**0.0392**	*1.57 *	**0.6419**	*25.68 *
Skinfolds (mm)							
Sk suprailiac	*18.7 *	**0.1970**	*3.68 *	***−*0.0095**	*−0.18 *	***−*0.1964**	*−3.67 *
Sk horiz. abdom	*20.0 *	**0.2350**	*4.70 *	***−*0.0105**	*−0.21 *	***−*0.2321**	*−4.64 *
Maturation (years)							
PHV	*−1.6 *	***−*0.6571**	*1.05 *	**0.0525**	*−0.08 *	**0.7047**	*−1.13 *

Total (kg)	Sum (FM) = 9.28			Sum (BMC) = 1.57		Sum (LST) = 28.40	

FM: fat mass, BMC: bone mineral content; LST: lean soft tissue; Sk: skinfold; PHV: age for peak height velocity. Against original values measured by DXA (FM = 9.30; BMC = 1.50; LST = 28.50).
